# Supplementary material for: Ellagic Acid Improves Antioxidant Capacity and Intestinal Barrier Function of Heat-Stressed Broilers via Regulating Gut Microbiota
Source: Animals (Basel). 2022 May 4;12(9):1180. doi: 10.3390/ani12091180 (PMC9131128; doi:10.3390/ani12091180)
Supplement: Supplementary file 1 [file animals-12-01180-s001.zip › animals-1663173-SI.pdf]

Supplementary Materials for:

Ellagic Acid Improves Antioxidant Capacity and Intestinal Barrier Function of Heat-Stressed Broilers via Regulating Gut Microbiota

Tai Yang [1], Bifan Liu [2], Yujie Wang [2], Xiangying Huang [2], Zhaoming Yan [2], Qian Jiang [2,*] and Qinghua Chen [2,*]

[1] Hunan Co-Innovation Center of Animal Production Safety, Changsha,410128, China; taiyang@stu.hunau.edu.cn
[2] College of Animal Science and Technology, Hunan Agricultural University, Changsha 410128, China; liubifan@stu.hunau.edu.cn (B.L.); wang2441645956@163.com (Y.W.); h2307604419@163.com (X.H.); yanzmmail@163.com (Z.Y.)
* Correspondence: jiangqian@hunau.edu.cn (Q.J.); chqh314@163.com (Q.C.); Tel.: +86-18570629932 (Q.J.); +86-15116320688 (Q.C.)

Table S1. Composition and nutrients of the basal diet (on air-dry basis).

**Items**	**Content**	
	**1 to 21 days of age**	**21 to 42 days of age**
Ingredient (%)		
Corn	56.22	63.13
Soybean meal	32.34	23.54
Fishmeal	3.92	6.76
$CaHPO_4$	1.46	0.78
Stone powder	1.10	0.95
Soybean oil	3.50	3.50
NaCl	0.30	0.30
Methionine	0.17	0.03
Premix[1]	1	1
Total	100	100
**Nutrition level[2]**		
ME (MJ/kg) [3]	12.54	12.96
CP (%)	21.50	20.00
Lysine (%)	1.20	1.12
Methionine (%)	0.5	0.40
Tryptophan (%)	0.25	0.22
Threonine (%)	0.82	0.77
Cystine (%)	0.33	0.30
Methionine +Cystine (%)	0.83	0.70
Ca (%)	1	0.9
Total P	0.72	0.65
Available P (%)	0.45	0.41

[1] Premix contains the following nutrients (per kilogram of diet): vitamin A, 10000 IU; vitamin D3, 2500 IU; vitamin B1, 3 mg; vitamin B2, 9 mg; vitamin B3(Niacin), 40 mg; vitamin B5, 15 mg; vitamin B6(Pyridoxine), 4 mg; vitamin B12,0.03 mg; vitamin E, 30 mg; vitamin K3, 1 mg; biotin, 0.20 mg; folic acid, 1 mg; Fe (ferrous sulfate), 120 mg; Cu (copper sulfate),10 mg; Zn (zinc sulfate), 100mg; Mn (manganese sulfate), 130 mg; I (potassium iodide), 0.7 mg; and Se (sodium selenite), 0.3 mg;
[2] Nutrition level is calculated according to NY/T33-2004.
[3] ME, metabolizable energy; and CP, crude protein.

Table S2. The analysis conditions for the measurements of serum biochemical parameters.

Items	Detection method	wavelength	Analysis type
Urea	Urease-GLDH, UV Method	340 nm	Kinetic method
glucose	HK Method	340 nm	endpoint method
total bilirubin	VOX Method	450 nm	endpoint method
total protein	Biuret Method	546 nm	endpoint method
total cholesterol	CHOD-POD Method	500 nm	endpoint method
triglycerides	GPO-POD Method	500 nm	endpoint method
aspartate aminotransferase	IFCC Method	340 nm	Kinetic method
alanine aminotransferase	IFCC Method	340 nm	Kinetic method
alkaline phosphatase	AMP Buffer Method	405 nm	Kinetic method
diamine oxidase	Spectrophotometry	340 nm	rate method
Lipopolysaccharide	Colorimetry	545 nm	Enzymatic method

Table S3. The sequences of primers used in this study.

**Genes**	**Primers Sequences**	**Product size**	**Gene bank no.**
β-actin	F: 5' ATGATGATATTGCTGCGCTCGT 3'<br>R: 5' CCCATACCAACCATCACACCT 3'	139 bp	NM_205518.2
ZO-1	F: 5' GCCTGAATCAAACCCAGCAA 3'<br>R: 5' TATGCGGCGGTAAGGATGAT 3'	182 bp	NM_001013611.2
Claudin-1	F: 5' ATGACCAGGTGAAGAAGATGC 3'<br>R: 5' TGCCCAGCCAATGAAGAG 3'	247 bp	NM_001244539.1
Nrf2	F: 5' CGCTTTCTTCAGGGGTAGCA 3'<br>R: 5' AGTTCGGTGCAGAAGAGGTG 3'	171 bp	NM_205117.2
NQO1	F: 5' GTTCAATGCCGTGCTCTCAC 3'<br>R:5' CCGCTTCAATCTTCTTCTGC 3'	146 bp	NM_001277620.2
HO-1	F: 5' GGTCCCGAATGAATGCCCTTG 3'<br>R: 5' ACCGTTCTCCTGGCTCTTGG 3'	137 bp	NM_205344.2
Gpx	F: 5' CTGCCTCCCACTTTCTCTTG 3'<br>R: 5' TCAAAGGGGCTCCCTAGTTT3'	215 bp	NM_001277853.3
CAT	F: 5' TATCAGAGGGACGGGCCAAT 3'<br>R: 5' GCACTACTGAAACGCTGCAC 3'	149 bp	NM_001031215.2
MnSOD	F:5' TACAGCTCAGGTGTCGCTTC 3'<br>R: 5' GCGAAGGAACCAAAGTCACG 3'	153 bp	NM_204211.2

Notes: ZO-1, Zonula occludens 1; Claudin-1; Nrf2, nuelear factor-E2-related factor 2; HO-1, heme oxygenase 1; NQO1, NAD(P)H:quinoneoxidoreductase; GPX1, glutathione peroxidase 1. CAT, catalase; MnSOD, manganese-containing superoxide dismutase.

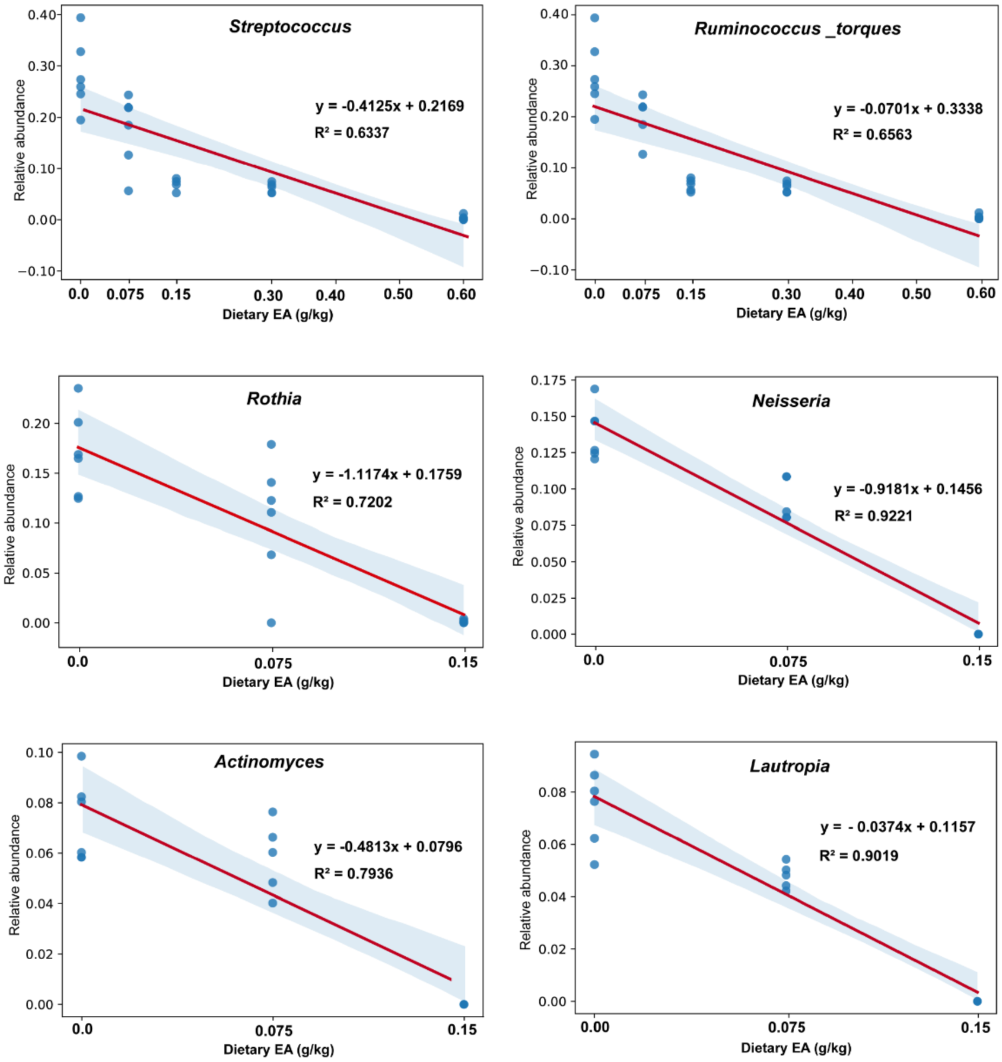

Figure S1. Linear-regression analysis between dietary EA dosage and cecum bacterial abundance. The broilers in the 5 experimental groups were supplemented with basal diets containing different levels of EA (0, 75, 150, 300, and 600 mg/kg) at HS temperature (35 ± 2°C), whose specific cecum microbiota abundance and dietary EA levels are represented at y-axis and x-axis, respectively.

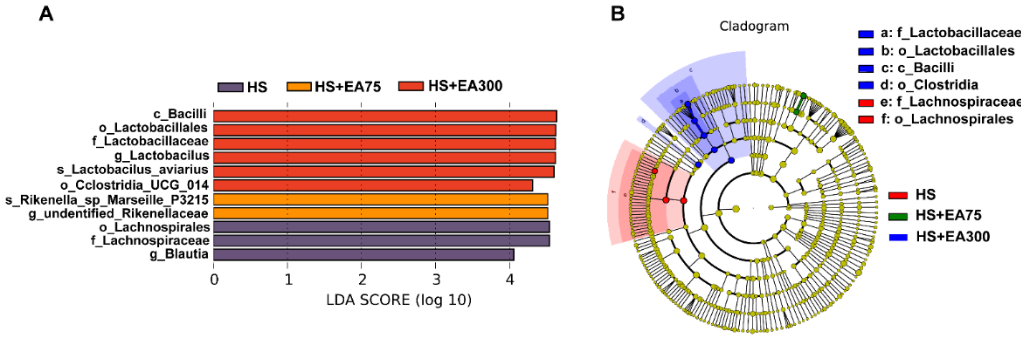

Figure S2. (A) LEfSe analysis of composition changes of cecum microbiota in heat stress (HS) group and HS + EA (75 and 300 mg/kg) group. (B) Cladogram plot of biomarkers of HS group and HS + EA (75 and 300 mg/kg) group. The broilers in the 5 experimental groups were supplemented

with basal diets containing different levels of EA (0, 75, 150, 300, and 600 mg/kg) at HS temperature (35 ± 2°C), which are represented as HS, HS+EA75, HS+EA150, HS+EA300, and HS+EA600, respectively.

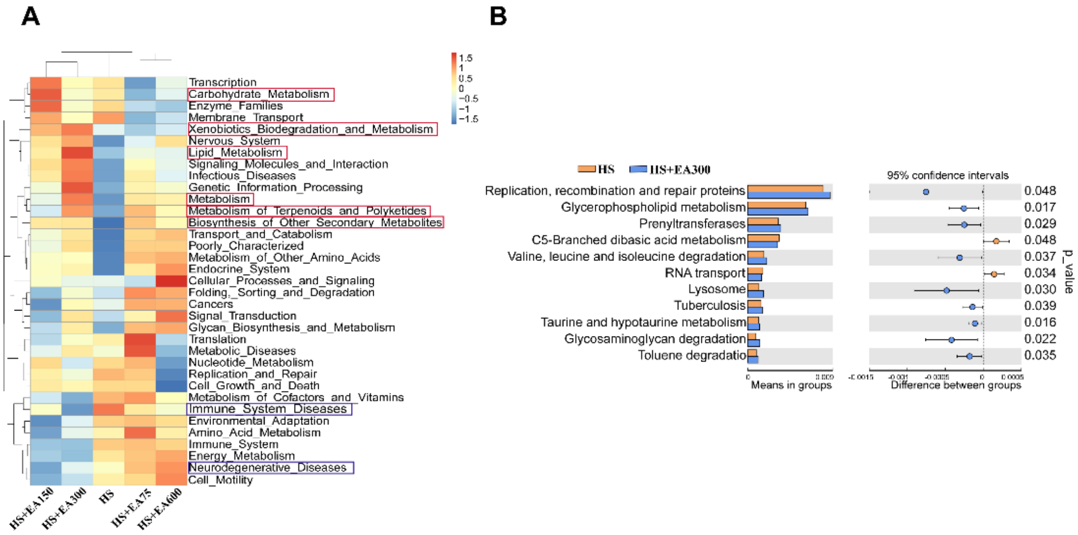

Figure S3. (A) Heatmap of the predicted functions of cecum microbial community based on PICRUSt analysis. (B) The Tax4Fun-based predicted functions of microbial community were compared between the heat stress (HS) group and the HS + dietary EA (300 mg/kg) group. The red box represents dietary EA supplementation increased the bacterial functions of carbohydrate metabolism, xenobioyics_biodegraddation_and metabolism, lipid metabolism, metabolism, metabolism of terprnoids and polyketides, biosynthesis of other secondary metabolites. The purple box represents increased bacterial functions of immune_system_ disease and neurodegeneration_disease.
